# *Bacillus velezensis* LMY3-5 for the biocontrol of soft rot in kiwifruit: antifungal action and underlying mechanisms

**DOI:** 10.3389/fmicb.2025.1562366

**Published:** 2025-04-29

**Authors:** Chunguang Ren, Yu Liu, Wenwen Su, Bing Tian

**Affiliations:** ^1^Guizhou Institute of Mountain Resources, Guiyang, Guizhou, China; ^2^Guizhou Agricultural Ecology and Resource Protection Station, Agriculture and Rural Affairs Department of Guizhou Province, Guiyang, Guizhou, China

**Keywords:** kiwifruit soft rot, *Botryosphaeria dothidea*, *Bacillus velezensis*, antifungal activity, biocontrol

## Abstract

Soft rot caused by *Botryosphaeria dothidea* is a serious fungal disease in kiwifruit observed during the storage period, and it seriously restricts the healthy and stable development of the kiwifruit industry. In the present study, the bacterial strain LMY3-5 with high antifungal activity was isolated from healthy kiwifruit tissues. Based on gyrA and 16S rRNA sequences, a phylogenetic tree was constructed, and LMY3-5 was identified as *Bacillus velezensis*. The 16% cell-free supernatant (CFS) of LMY3-5 suppressed *B. dothidea* spore germination and mycelial growth by 97.32%. The 16% CFS of LMY3-5 could effectively inhibit the expansion of kiwifruit soft rot *in vitro*, and the inhibition rate was 73.59%. The scanning electron microscopy (SEM) observations of *B. dothidea* mycelia treated with the 16% CFS for 12 h showed that the mycelia were curved, wrinkled, and sunken. Moreover, transmission electron microscopy (TEM) observations revealed blurred boundaries of mycelial cell walls as well as plasmolysis and vacuolization. Propidium iodide (PI) staining showed that the CFS treatment damaged the cell membrane of *B. dothidea* and affected its permeability, which led to leakage of the nucleic acids and proteins. Simultaneously, a substantial increase in the activity of key enzymes (i.e., *β*-1,3-glucanase and chitinase) was observed, which indicated damage to the cell wall function of *B. dothidea.* GC/LC–MS analysis revealed the presence of 27 antimicrobial compounds. Thus, the LMY3-5 strain showed great potential as a biocontrol agent for soft rot disease in kiwifruit.

## 1 Introduction

Kiwifruit is a commonly consumed fruit worldwide that originated in China (Huang, 2016), and it is rich in nutrients and contains a variety of essential amino acids, dietary fibers, minerals, and vitamins (e.g., vitamin C, vitamin E, and vitamin K) (Aneta and Paulina, 2018; Lian et al., 2019; Wang et al., 2022; Wojdylo et al., 2017; Zhu et al., 2019). Kiwifruit is widely cultivated in many countries, such as New Zealand, China, Chile, and Italy (Nazir et al., 2024), and the growth of the kiwifruit industry has led to annual increases in its planting area. However, various diseases have become increasingly prominent with expansion in the production scale. They include kiwifruit canker (Kim et al., 2019), soft rot (Guo et al., 2024), crown gall (He et al., 2022), bloom blight (Balestra et al., 2009), and leaf spot disease (Chen et al., 2022).

Kiwifruit soft rot is a major disease that affects production in China (Li et al., 2017), Korea (Kwon et al., 2011), Italy (Luongo et al., 2011), and Chile (Diaz et al., 2014). The key pathogens responsible for soft rot in kiwifruit are the fungi *Diaporthe eres* and *Botryosphaeria dothidea* (Diaz et al., 2017; Zhou et al., 2015). The disease mainly affects fruit during storage and manifests as changes in the color of the fruit, which turns from yellowish green to dark green, and the texture that becomes soft, rotten, and concave. Spraying chemical antifungal agents such as carbendazim and tebuconazole before harvest is a practical and inexpensive method of preventing kiwifruit decay (Kim et al., 2013; Shin et al., 2021). However, long-term application of chemical antifungal agents may induce drug fungicide resistance in pathogens; in addition, excessive pesticide residues can cause environmental pollution and public health problems (Bakirci et al., 2014; Yin et al., 2023). Increasing awareness of food safety and environmental protection demand environmentally friendly, efficient, and safe disease prevention and control measures.

Biological control agents have emerged as an efficient and environmentally safe method of preventing pathogen infections in fruits after harvest (Jongman et al., 2022; Shi et al., 2024; Torres-Palazzolo et al., 2024). The most successful and widely used biocontrol bacteria are *Bacillus* spp., which can synthesize antibacterial molecules, including bacteriocins, cell wall-disintegrating enzymes, lipopeptide antibiotics, and peptides (Hamdache et al., 2011; Awad et al., 2012). Lipopeptides can destroy the membranes and walls of fungal cells, thus causing intracellular content leakage (Cawoy et al., 2015; Zou et al., 2018). In addition, *Bacillus* may synthesize growth-promoting substances, such as auxins, gibberellins, indole-3-acetic acid (IAA), and cytokinins (Meng et al., 2016; Kanjanamaneesathian et al., 2013). The antifungal activity of *Bacillus* may be exerted via the release of microbial agents, which can be used in the preparation of antifungal films and microbial fertilizers. Moreover, these bacteria can also effectively control diseases in vegetables and fruits after harvest.

In this study, we isolated a strain of *Bacillus* (LMY3-5) from “Guichang” kiwifruit that showed strong antifungal activity against *B. dothidea*. The purpose of this study was to (1) identify the species of *Bacillus* strains; (2) analyze the *in vitro* and *in vivo* antifungal properties and antifungal mechanisms of the LMY3-5 cell-free supernatant (CFS); and (3) detect the antifungal metabolites of strain LMY3-5.

## 2 Materials and methods

### 2.1 Microorganisms

In 2023, strain LMY3-5 was isolated from kiwifruit in the molecular laboratory of the Guizhou Provincial Institute of Mountain Resources, and it has since been preserved in the Chinese Microbial Strain Storage Management Center (CGMCC 29700). Strain LMY3-5 was grown on 100 mL Luria-Bertani (LB) medium at 30°C and 200 rpm for 12 h to prepare the inoculum.

Five plant pathogenic fungi, *B. dothidea, Diaporthe eres*, *Monilinia fructicola*, *Alternaria alternata*, and *Colletotrichum gloeosporioides*, were isolated and identified at the molecular laboratory of Guizhou Provincial Institute of Mountain Resources. The pathogens were grown on potato dextrose agar (PDA) medium at 25°C for 5–7 days.

### 2.2 *In vitro* antagonism assay

The antagonistic activity of strain LMY3-5 against five pathogens was tested by using a dual culture technique (Yan et al., 2020). Mycelial discs (5 mm diameter) of *B. dothidea*, *D. eres*, *M. fructicola*, and *A. alternata* were placed in the middle of fresh PDA plates (90 mm). Strain LMY3-5 was inoculated at a distance of 2 cm from the mycelial disc of the fungal pathogen. Control plates were inoculated only with the mycelial discs of fungal pathogens. All experiments were carried out three times, and each treatment included three replicates of the number of plates. All media plates were kept in an incubator for 5 days at 28°C. Subsequently, the colony size (diameter) was measured to calculate the inhibition rate.

Inhibition rate (%) = [(dcontrol − dtreatment) / dcontrol] × 100.

### 2.3 Identification of strain LMY3-5

Strains LMY3-5 were streaked on the LB plate and cultured overnight in an incubator at 37°C. The plate was observed to determine the shape, color, size, and other characteristics of the colonies. The colonies were subjected to Gram staining and observed and imaged under a Leica DM4 B microscope.

Microbial DNA was isolated from each antifungal strain utilizing a Biomiga Bacteria DNA extraction kit (Sangon Biotech Co., Ltd.) and employed as the PCR template. 16S rRNA (Weisburg et al., 1991) and gyrA (Chun and Bae, 2000) sequences of the bacterial strains were amplified using PCR primers 27F/1492R and gyrA-42f/gyrA-1066r, respectively, and the obtained amplicons were outsourced for sequencing to Sangon Bioengineering Co., Ltd. (Shanghai, China). The sequencing results were analyzed by drawing comparisons against the GenBank database, and highly homologous sequences were downloaded as reference sequences. Bayesian inference (BI) and maximum likelihood (ML) techniques were employed using CIPRES ScienceGateway V.3.3 (Miller et al., 2010) to prepare a phylogenetic tree. The obtained tree file was viewed and resized in FigTree v.1.4.0 and exported as a PDF file.

### 2.4 Impact of bacterial cell-free supernatant on the growth of pathogenic fungi

The cell-free supernatant (CFS) was prepared using protocols given in a previous study (Liu et al., 2023). Strain LMY3-5 was cultured in LB medium at 30°C and 200 rpm for 1 day and used as the seed culture. Then, 5 mL of seed culture was further cultured in LB medium (100 mL) at 30°C and 200 rpm for 3 days. The supernatant was subjected to centrifugation (12,000 rpm for 15 min) and filtration (0.22 μm sterile filter) to obtain the CFS. The obtained CFS was mixed with molten PDA medium (45 ± 5°C) to a final concentration of 1, 2, 4, 8, and 16% (vol/vol) and poured into sterile petri dishes. PDA plates without the CFS were used as controls. The fungal plug was placed at the center of the PDA plate and incubated at 25°C for 3 days. All experiments were carried out three times, and each treatment included three replicates of the number of plates. After measuring the colony size, the inhibition rate was determined as discussed in section 2.1.

### 2.5 Influence of CFS on spore germination of *B. dothidea*

Conidia formed on the *B. dothidea* plate were scraped and diluted with sterile water to a concentration of 1 × 10^6^ mL^−1^. The obtained CFS was mixed with the spore suspension to a final concentration of 0 (as control), 1, 2, 4, 8, and 16% (volume percentage of CFS in deionized Water) and shaking culture at 28°C, 120 rpm for 3 h, 6 h, 12 h. Spore germination in both groups was examined using an optical microscope. Germination was defined as an increase in the spore germ tube length beyond the spore radius. Each experiment was performed three times. After measuring the spore germination rates of both groups, the germination inhibition rate was calculated as follows:

Spore germination inhibition rate (%) = (germination rate of control / germination rate after treatment) / (germination rate of control) × 100.

### 2.6 Impact of CFS on kiwifruit soft rot

Fresh and healthy kiwifruits of the same size were selected, and their surfaces were disinfected. Three wounds were made on the surface of each kiwifruit using sterile toothpicks, and then the fruits were dipped into a solution containing 1, 2, 4, 8, and 16% of LMY3-5 CFS for 60 min. In the control group, the kiwifruits were dipped into in sterile water. Then, mycelia discs (5 mm diameter) of *B. dothidea* were placed on the kiwifruit wounds. Each treatment consisted of 20 kiwifruits, which were placed in plastic boxes maintained at 28°C and 90% relative humidity. After 7 days, the lesion diameters were estimated. Disease inhibition rate (%) was determined by the following equation: (A1 − A2) / A1 × 100, where A1 and A2 correspond to the lesion diameter of the control and treatment groups, respectively.

### 2.7 Morphological characteristics of *B. dothidea* mycelia

Fungal mycelial morphology was observed using SEM (SU-8010, Hitachi, Japan). Mycelia treated with CFS [16% (v/v)] for 12 h and fixed using 2.5% (v/v) glutaraldehyde at 4°C for 24 h. After discarding the fixative, the samples were rinsed for 15 min (3 times) with 0.1 M phosphate buffer (pH 7.2). Then, the samples were exposed to an ethanol gradient of 30, 50, 70, 80, 90, and 100% (20 min each). Subsequently, the dehydrated mycelia were critical-point-dried with CO_2_ and then coated with gold. After freeze-drying under vacuum, the samples were observed via SEM and images were acquired.

### 2.8 Structural characterization of *B. dothidea* cells

The cellular microstructure of *B. dothidea* was observed via TEM (EM1200EX, JEOL, Tokyo, Japan). Sample preparation steps were the same as those described in section 2.6. After sequentially drying in an ethanol gradient of 30, 50, 70, 80, 90, and 100%, the samples were treated for 20 min with pure acetone. The samples were then embedded with Epon812 fixative, cut into ultrathin sections, sequentially stained with uranium acetate and lead citrate, and observed and imaged via TEM.

### 2.9 Propidium iodide (PI) staining determination

Three fresh mycelia discs of *B. dothidea* were inoculated in 100 mL potato dextrose broth (PDB) and cultured on a rotary shaker (150 × g at 25°C) for 3 days. Then, the mycelia were harvested using two layers of sterile coarse cotton cloth and washed three times with sterile water. The wet mycelia were then transferred to fresh PDB at final CFS concentrations of 0% (as control), 4, 8, and 16% (vol/vol) and cultured for 12 h. The control group only contained PDB-cultured hyphae. The mycelia were collected and stained with 1 mg/mL PI at 25°C in dark for 30 min, and then excess dye solution was washed off with PBS. Images were captured using confocal fluorescence microscopy (NE 910-FL, Ningbo Yongxin Optics Co., Ltd., China).

### 2.10 Detection of intracellular content leakage

The mycelia samples of *B. dothidea* were prepared according to the above-described method. The same amount of mycelium (1 g) was weighed and resuspended in PDB containing 4, 8, and 16% (volume ratio) of CFS. The control group only contained PDB-cultured hyphae. The supernatant was collected by centrifugation at 8000 × g for 5 min after culturing for 0.5 h, 1 h, 3 h, 6 h, and 12 h. Subsequently, 200 μL supernatant was placed on a multifunctional microplate (SuperMax 3,100, Shanghai Flash Biotechnology Co., Ltd., China) to record the absorbance at 280 nm and 260 nm. Each treatment was performed three times to calculate the relative leakage of proteins and nucleic acids (Liu et al., 2020; Li et al., 2021).

### 2.11 Activity levels of chitinase and *β*-1,3-glucanase

The mycelia samples of *B. dothidea* were prepared according to the above-described method. The same amount of mycelia (1 g) was resuspended in PDB medium containing 4, 8, and 16% (volume ratio) CFS for 3 h, 6 h, 12 h, 24 h, 48 h, and 72 h. Each treatment was conducted in triplicate. Chitinase activity assay kit (BC0820, Beijing Solarbio Science and Technology Co., Ltd., China) and *β*-1,3-glucanase activity assay kit (BC0360, Beijing Solarbio Science and Technology Co., Ltd., China) were used according to the manufacturer’s instructions, and the activities of chitinase and *β*-1,3-glucanase in *B. dothidea* cells were measured using spectrophotometry.

### 2.12 Analysis of filtrate components via GC–MS and LC–MS

The fermentation broth of LMY3-5 cultured for 7 days was filtered (0.22 μm membrane). Components of the CFS filtrate were assessed by GC-LC–MS. GC–MS (Agilent Technologies, CA, USA) was used to identify the CFS components using a DB-5MS capillary column (membrane width, 0.25 μm; 30 m × 0.25 mm) (Li et al., 2021). LC–MS analysis was performed as elaborated by Nam et al. (2015) (API2000 TM, AB Sciex, Redwood City, CA, USA) and eluted by employing an acetonitrile/water mobile phase consisting of 0.05% TFA. The MS instrument included an electrospray ionization (ESI) source and triple quadrupole spectrometer.

### 2.13 Statistical analyses

All data were statistically analyzed using Excel 2010 and SPSS version 25 (SPSS Inc., Chicago, IL, USA). One-way ANOVA followed by Duncan’s multiple range test was performed to determine significant differences at *p* < 0.05. Charts were plotted with Origin 2021.

## 3 Results

### 3.1 Antifungal activity of LMY3-5 against phytopathogenic fungi

**Figure 1:**
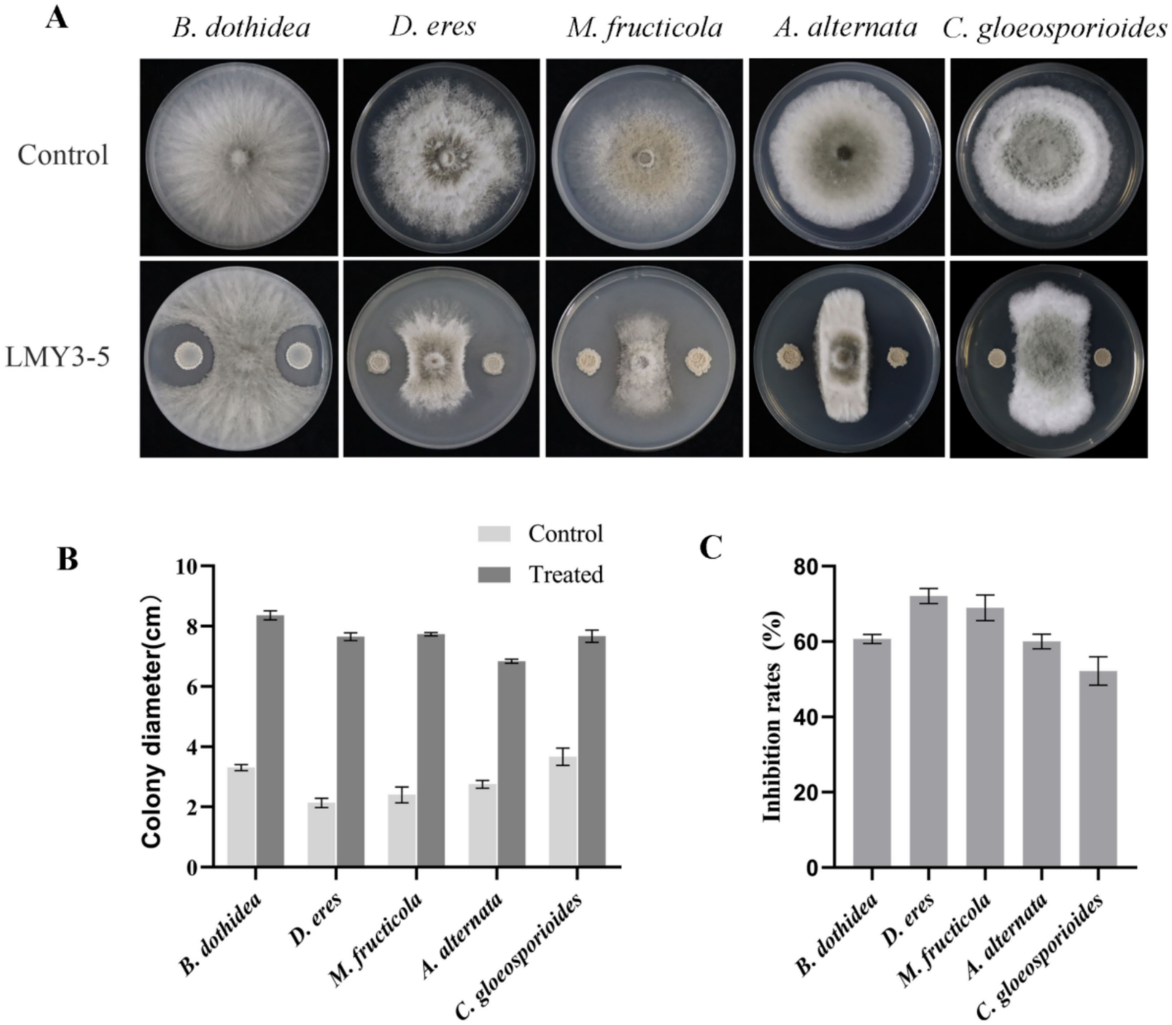
Antifungal activity of strain LMY3-5 against phytopathogenic fungi. **(A)** Mycelial growth of phytopathogenic fungi under the LMY3-5 treatment. Colony diameter(cm) **(B)** and Inhibition rate **(C)** of strain LMY3-5 against pathogenic fungi. Data are presented as the means ± SD [*n* (number of plates) = 3].

Strain LMY3-5 showed strong inhibitory activity. In the confrontation culture experiment, the inhibition rates of *D. eres*, *B. dothidea*, *A. alternata, M. fructicola,* and *C. gloeosporioides* against mycelial growth were 72.11, 60.68%, 60.05, 68.97 and 52.18%, respectively (Figure 1).

### 3.2 Identification of strains LMY3-5

**Figure 2:**
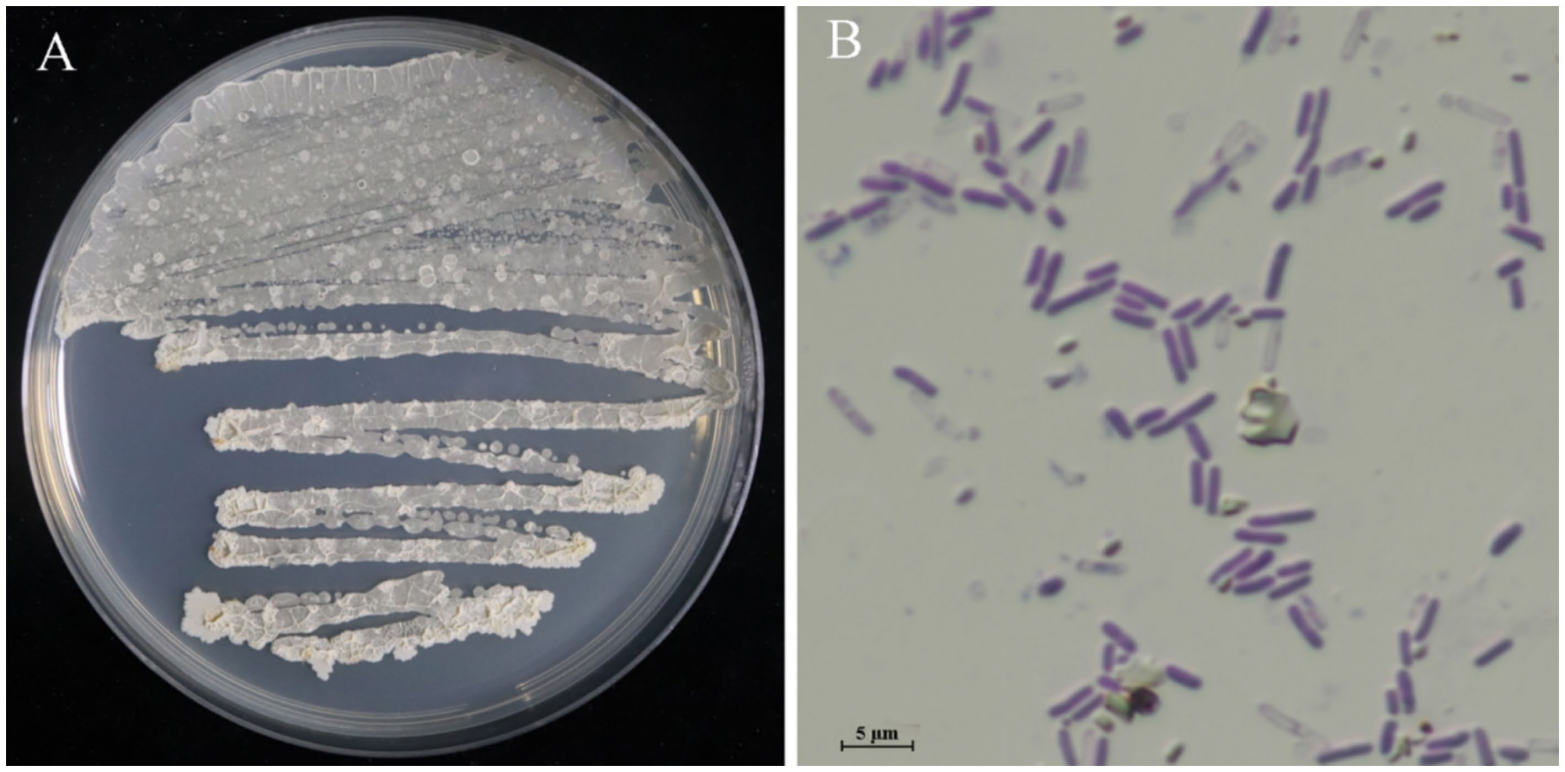
Colony morphology of strain LMY3-5. **(A)** Colony morphology in LB; **(B)** Gram staining results.

**Figure 3:**
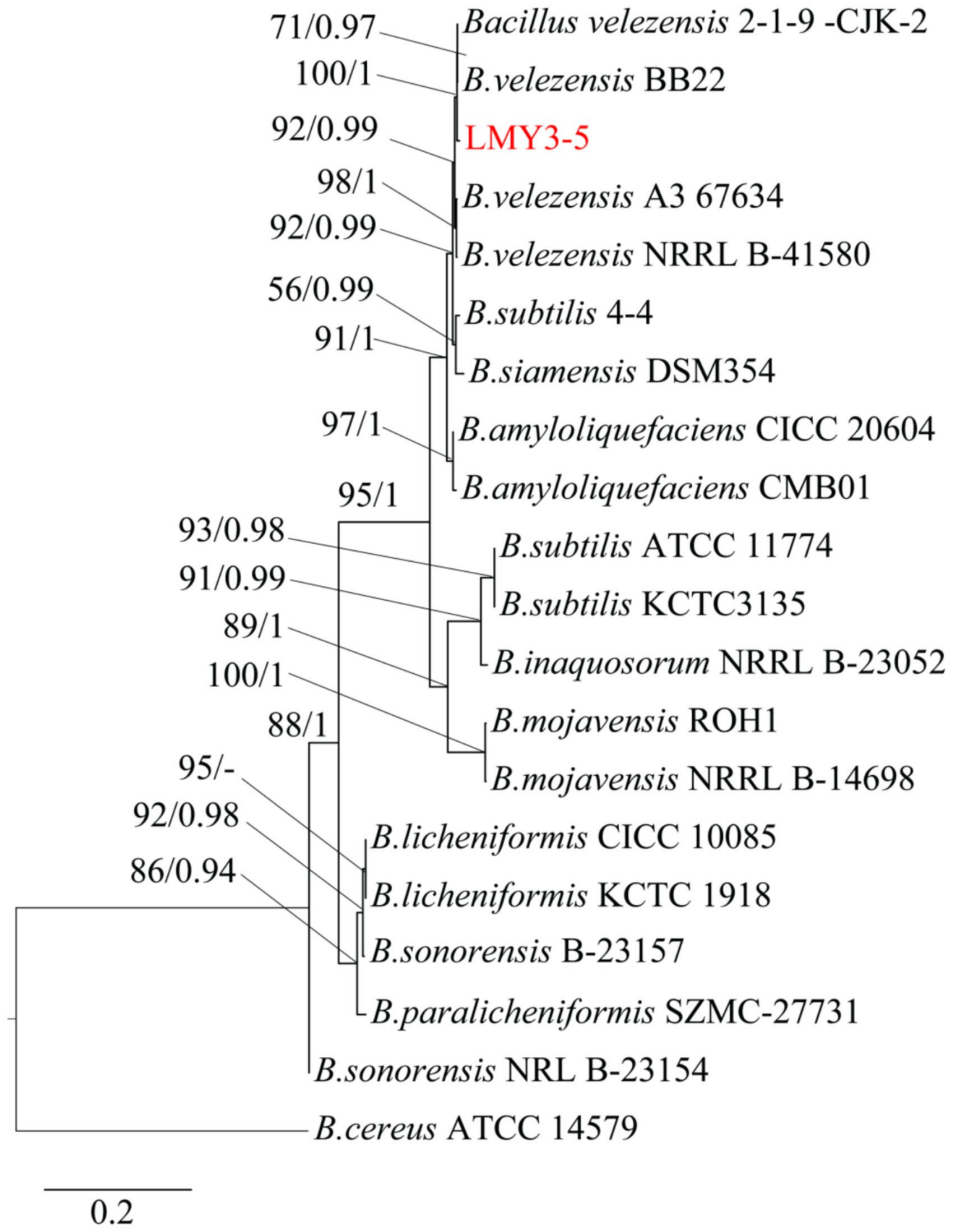
Maximum likelihood tree based on the gyrA and 16 S rRNA sequences. Based on 1,000 bootstrap repetitions, the BYPP support rate was >0.90 and ML value was >70%, as shown at the node.

After culturing for 3 days on solid LB, strain LMY3-5 produced milky white colonies with rough and wrinkled surfaces and irregular and opaque edges (Figure 2A). When stained with Gram stain, LMY3-5 retained the purple color, indicating its Gram-positive nature (Figure 2B). To further determine the taxonomic status of LMY3-5, we identified it at the molecular level. The gyrA and 16 S rRNA sequences of LMY3-5 strain were stored in the NCBI database (accession numbers PP239377 and PP231028, respectively). BLAST alignment analysis was performed on the 16 S rRNA and gyrA genes sequenced, and the corresponding sequences of 19 *Bacillus* strains with high homology were selected as reference sequences for multi-gene phylogenetic analysis. The phylogenetic tree is shown in Figure 3. Strains LMY3-5 and *B. velezensis* were clustered in one branch, and the support rate was 100/1. Based on morphological and molecular systematic results, strain LMY3-5 was identified as *B. velezensis*.

### 3.3 *B. dothidea* mycelial growth suppression by CFS

**Figure 4:**
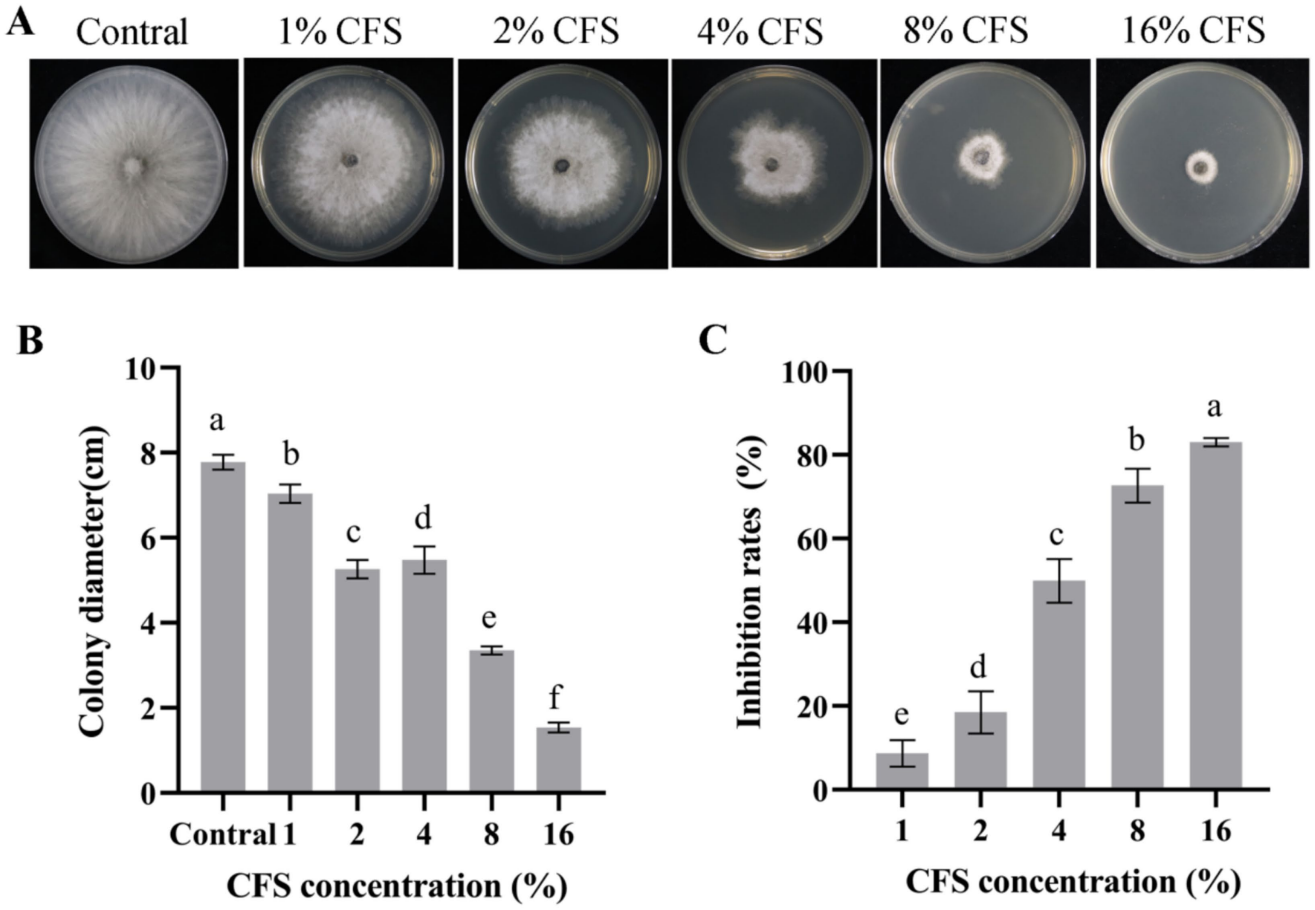
Antifungal effect of strain LMY3-5 on *B. dothidea* determined using different concentrations of CFS *in vitro*. **(A)** Mycelial growth of *B. dothidea* on PDA under CFS treatment. Colony diameter(cm) **(B)** and mycelial inhibition rate **(C)** of *B. dothidea* under CFS treatment. Data are presented as the means ± SD [*n* (number of plates) = 3]. Bars with the same lowercase letters indicate no significant treatment results (*p* < 0.05).

The CFS of LMY3-5 substantially suppressed the expansion of and *B. dothidea* mycelia (Figure 4). The antifungal activity increased rapidly as the concentration of CFS increased. When the amount of CFS reached 16%, the inhibition rate of *B. dothidea* reached 83.51% (Figure 4C).

### 3.4 Impact of LMY3-5 on the germination of *B. dothidea* spores

**Figure 5:**
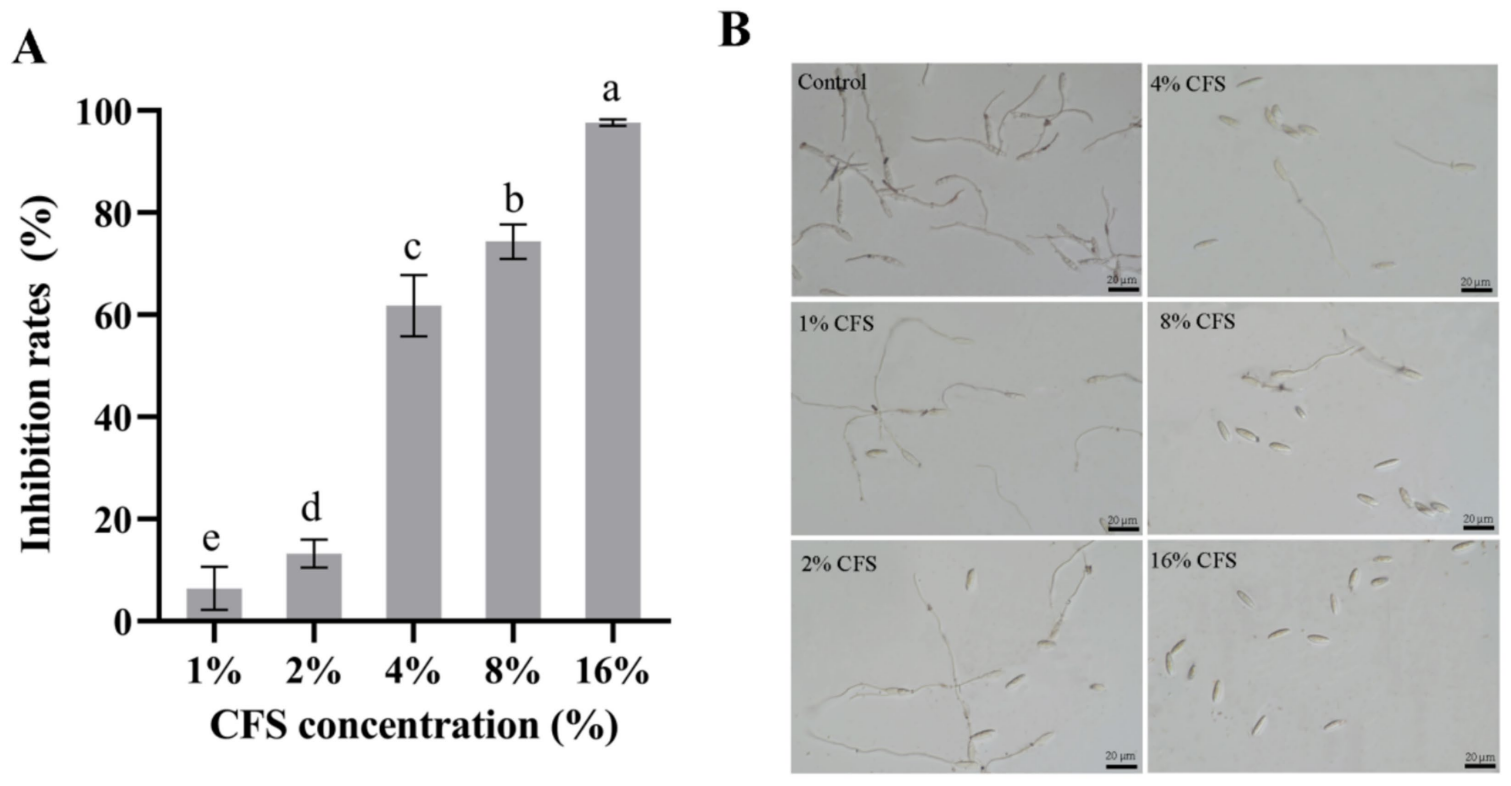
Inhibitory effect of LMY3-5 CFS on *B. dothidea* spore germination. **(A)** Inhibition rate of *B. dothidea* spore germination under CFS treatment **(B)** Spore germination of *B. dothidea* inhibited by CFS were observed under microscopy. Data are presented as the means ± SD. Bars with the same lowercase letters indicate no significant difference between treatments (*p* < 0.05).

Different concentrations of CFS of the antagonistic strain LMY3-5 were used to detect the suppression of germination of *B. dothidea* spores. Addition of the supernatant at 16.00% (v/v) led to the highest inhibition by CFS (Figure 5A) relative to that of the control. The rate of spore germination inhibition was 97.32% (Figure 5A). In the 8.00% (v/v) and 4.00% (v/v) CFS treatment groups, the spore germination inhibition rates were 72.76 and 53.65%, respectively. Overall, the CFS of LMY3-5 successfully suppressed the germination of *B. dothidea* spores. Stronger suppression was observed as the CFS concentration in PDB increased.

### 3.5 Inhibition of soft rot development in kiwifruit by LMY3-5

**Figure 6:**
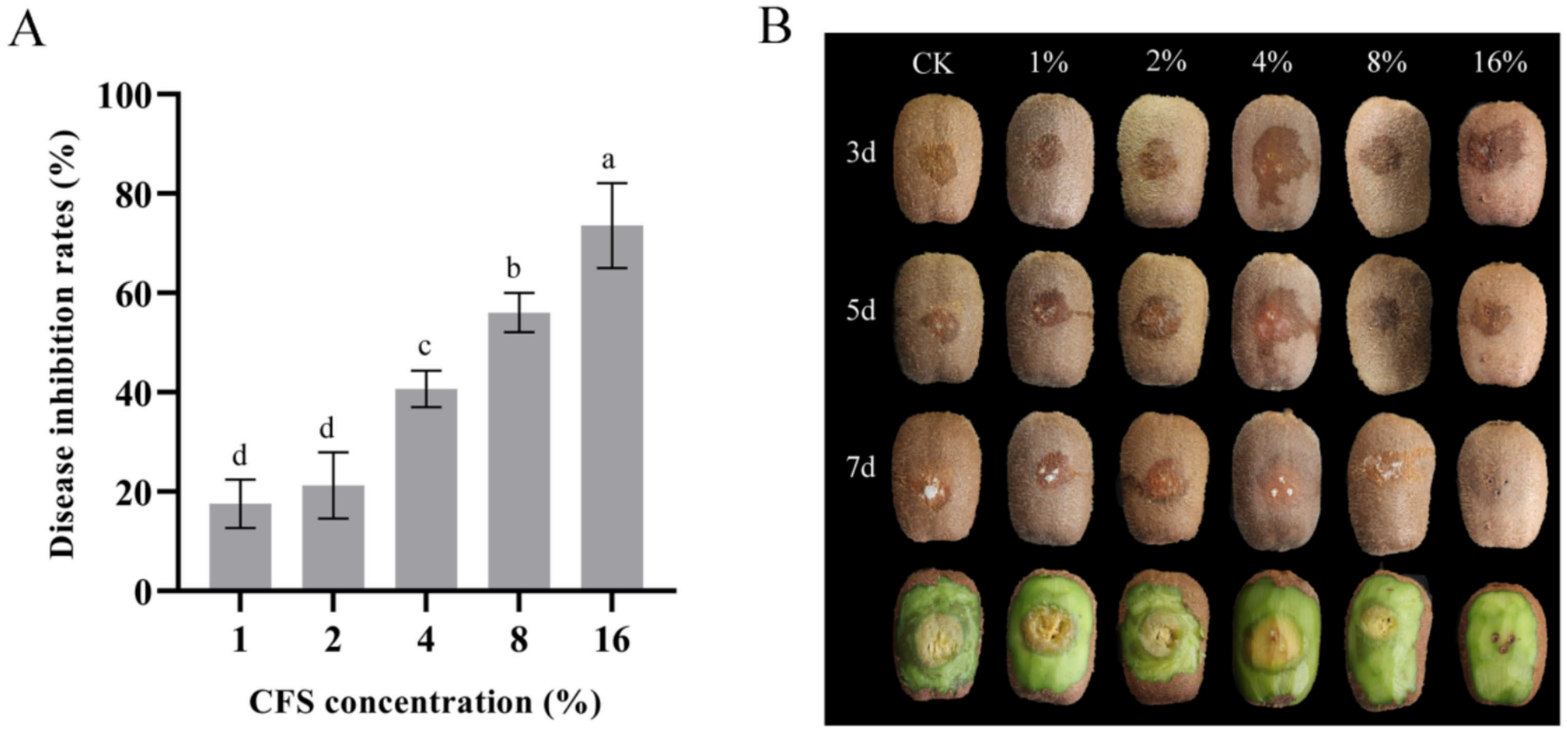
*In vivo* effect of LMY3-5 CFS on disease extension in kiwifruit caused by *B. dothidea*. **(A)** Disease inhibition rate after treatment with different concentrations of aseptic filtrates. **(B)** Disease extension symptom in kiwifruit after treatment with LMY3-5 CFS for 3, 5, and 7 days. Data are presented as the means ± SD [*n* (number of kiwifruit) = 20]. Bars with the same lowercase letters indicate no significant difference between treatments (*p* < 0.05).

To detect the control effect of strain LMY3-5 on kiwifruit soft rot, kiwifruits were exposed to *B. dothidea*. After 1 week of treatment, the kiwifruits were peeled and observed. The kiwifruit in the control group without the CFS was severely diseased, the lesion diameter was large, and the fruit was rotten. However, kiwifruits in the CFS treatment groups showed small lesion diameters. In addition, the greater the concentration of CFS, the smaller the lesion diameter and better the inhibitory effect (Figure 6). The inhibition rates of kiwifruit inoculated with *B. dothidea* treated with 16% CFS were 73.59% (Figure 6A). LMY3-5 could efficiently inhibit infection by *B. dothidea,* slow the expansion of lesions, and exert a significant control effect on soft rot in kiwifruit.

### 3.6 Effects of LMY3-5 on *B. dothidea* mycelial morphology

**Figure 7:**
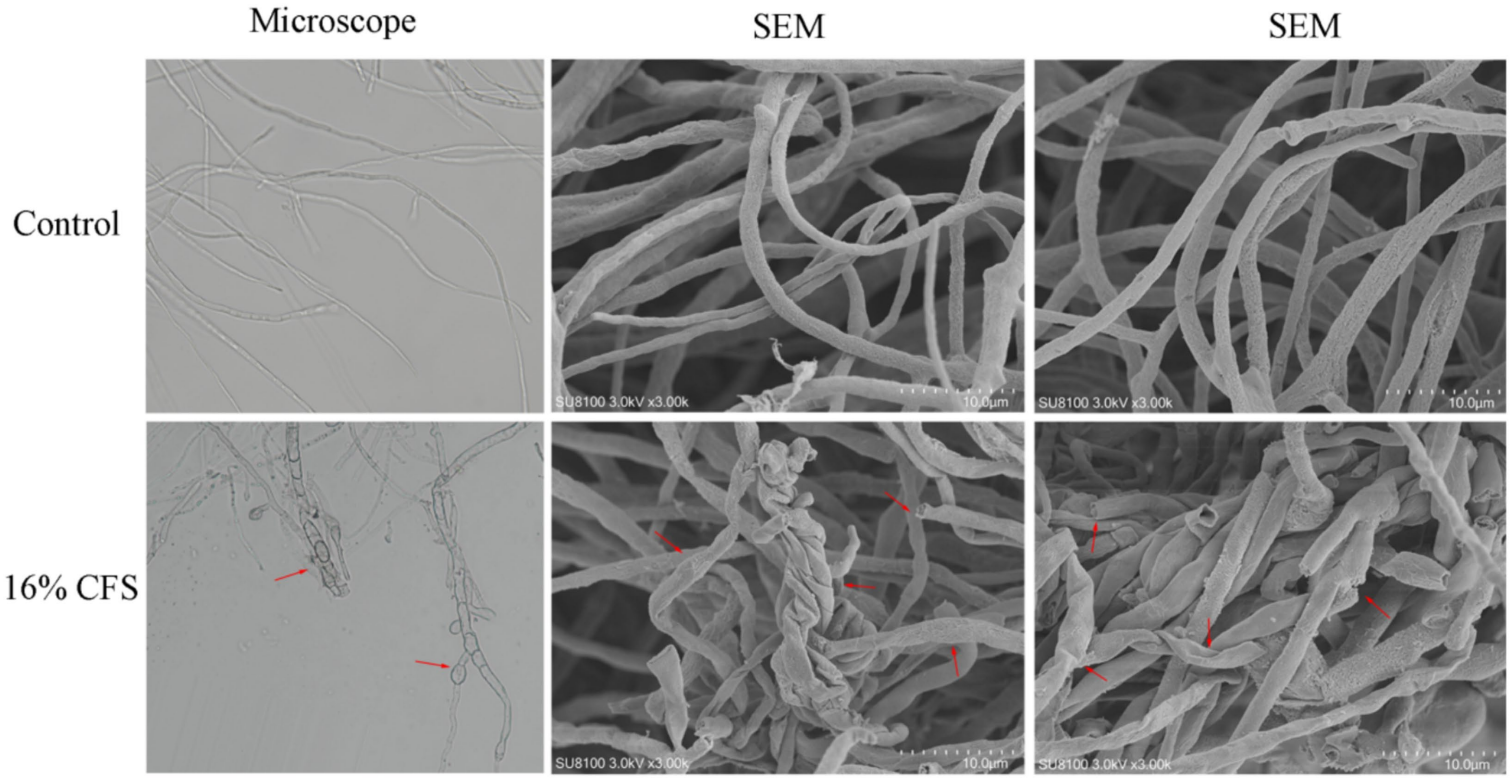
Effect of 16% CFS treatment on *B. dothidea* mycelium morphology after 12 h. Control was treated with sterile water for 12 h. The red arrow indicates collapse, shrinkage, or mycelial fracture.

Morphological variations between the mycelia of untreated and treated *B. dothidea* were assessed using SEM. The surface of the control mycelia was full and complete and did not show shrinkage or depressions, and the thickness was uniform (Figure 7). After treatment with the CFS, the mycelia showed abnormalities in surface morphology as well as collapse, shrinkage, and mycelial fracture.

### 3.7 Impact of LMY3-5 on the structure of *B. dothidea*

**Figure 8:**
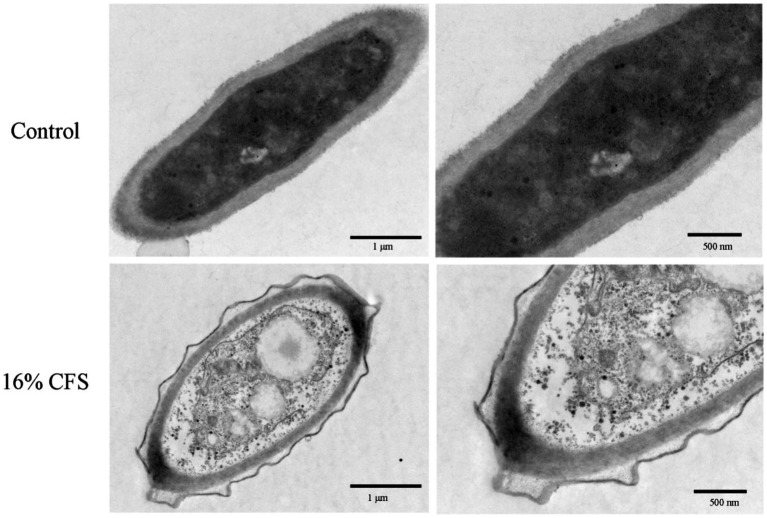
Transmission electron microscope scans of *B. dothidea* mycelia treated with 16% CFS for 12 h. Control was treated with sterile water for 12 h.

Structural variations in *B. dothidea* cells exposed to LMY3-5 were examined by TEM. Untreated mycelial cells were healthy, the cell membrane and cell wall were normal in shape, and the organelle contours were clear and evenly distributed in the cytoplasm (Figure 8). In treated mycelia, the cell structure was incomplete, cell walls were blurred, cellular membranes were contracted, internal structure was loose, organelles were severely dissolved, and a large cavity area appeared.

### 3.8 Effect of CFS on the cell membrane of *B. dothidea*

**Figure 9:**
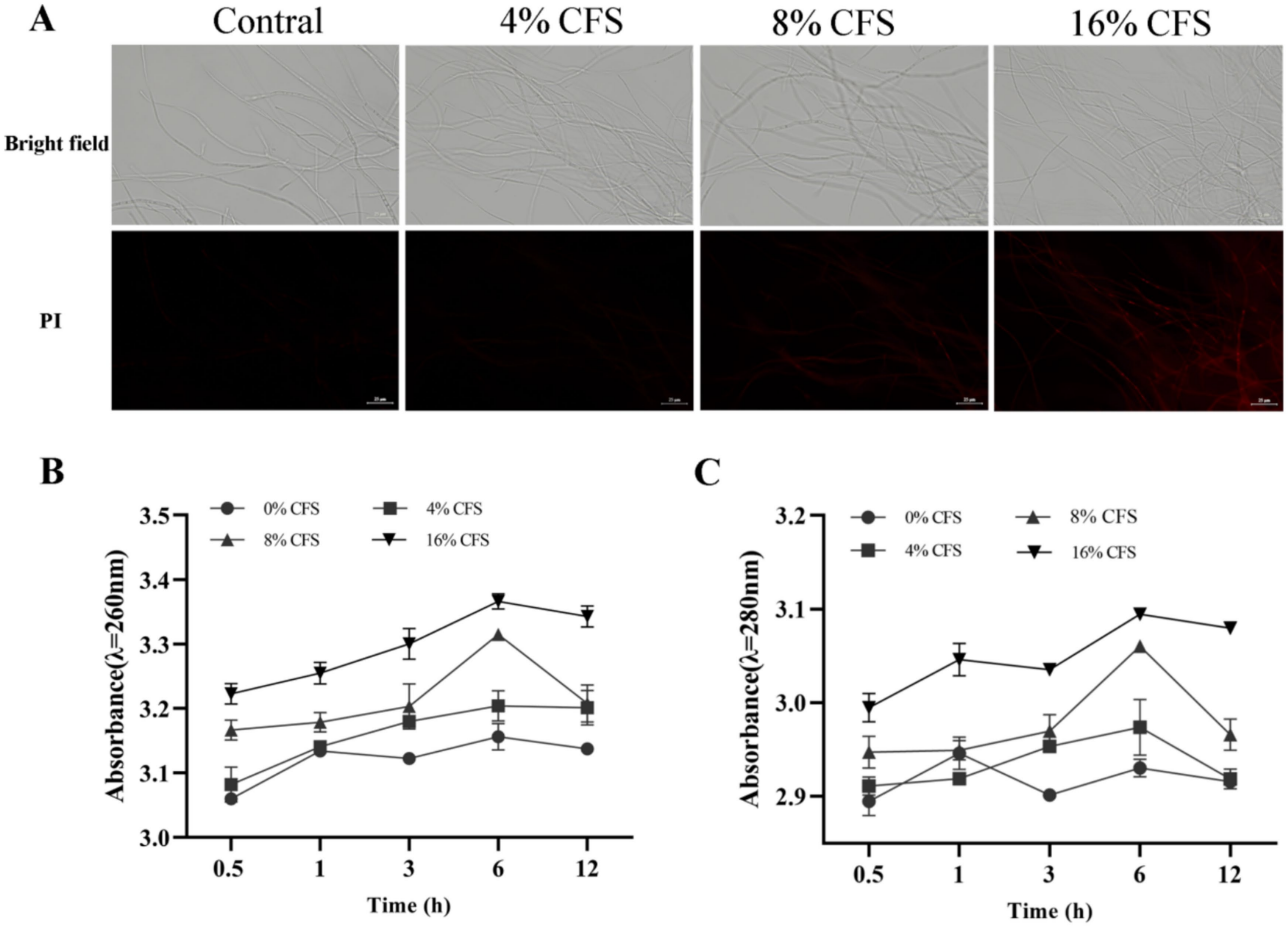
Effects of CFS of *B. velezensis* LMY3-5 on cell membrane integrity of *B. dothidea*. **(A)** Images of propidium iodide-stained cells in the dark field (PI) and bright field. **(B)** Concentrations of leaked nucleic acids and **(C)** proteins of *B. dothidea* under CFS treatment were measured at various time points. Data are presented as the means ± SD. Bars with the same lowercase letters indicate no significant difference between treatments (*p* < 0.05).

PI is able to stain the nucleus through the cell membrane of dead cells and middle and late apoptotic cells, thereby directly indicating the extent of damage to the mycelial cell membrane. As shown in Figure 9A, after 12 h of CFS treatment, *B. dothidea* hyphae showed a red color via fluorescence microscopy, and the fluorescence intensity increased with increasing CFS concentration. The results showed the noticeable destruction of the *B. dothidea* cell membrane by LMY3-5.

Nucleic acids and proteins are important intracellular components that can be used to evaluate changes in the permeability of the mycelial membrane. The extracellular protein and nucleic acid content of *B. dothidea* increased with prolonged treatment time and increased CFS concentration, whereas the CK group remained the same (Figures 9B and C). After 6 h of CFS treatment, a high quantity of nucleic acids and proteins began to leak from the *B. dothidea* mycelia. CFS destroyed the cell membrane and improved its permeability, resulting in a dose-dependent increase in nucleic acids, proteins, and leakage.

### 3.9 Impact of LMY3-5 on the activity levels of cell-wall-disintegrating enzymes in *B. dothidea*

**Figure 10:**
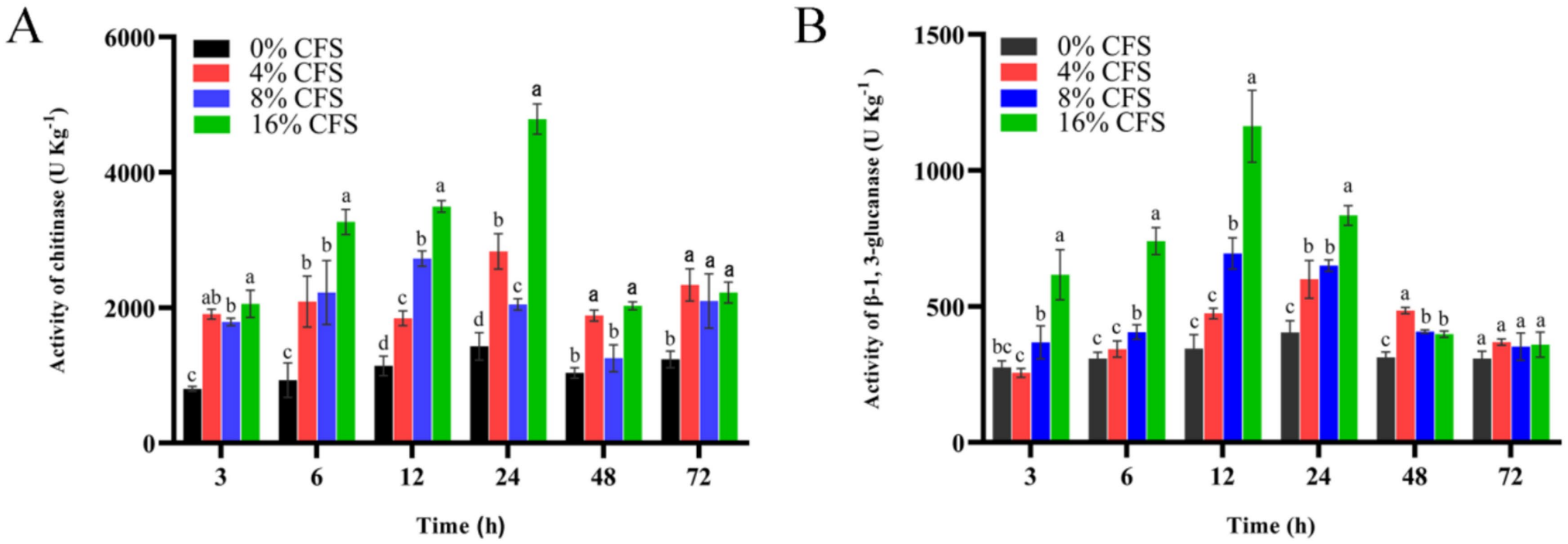
Impact of LMY3-5 on the chitinase **(A)** and glucanase **(B)** activities in *B. dothidea*. Data are displayed as the mean ± SE. Bars with the same lowercase letters indicate no significant difference between treatments (*p* < 0.05).

Chitinase and *β*-1,3-glucanase are key cell wall-degrading enzymes, which are important indicators of cell wall integrity. Activity levels of these enzymes in LMY3-5-treated *B. dothidea* initially surged and then declined (Figure 10). Chitinase activity was highest after 12 h, while *β*-1,3 glucanase activity was highest after 24 h. At each measurement time point, the activity levels of these enzymes were noticeably greater in treated *B. dothidea* relative to that of the control. This shows that the CFS decreased the activity of key enzymes in the cell wall, thus leading to a gradual degradation of the cell wall.

### 3.10 GC–LC–MS identification of CFS components

A total of 181 volatile components were identified from LMY3-5 CFS by GC–MS. A literature review revealed that 10 compounds to have antibacterial or antifungal activity (Table 1). The LC–MS results showed that a total of 2,901 compounds were detected, of which 204 components had similarity >80% and 17 metabolic components showed antibacterial or antifungal activity (Table 2).

**Table 1:** Antimicrobial active components identified from CFS of LMY3-5 by GC–MS analysis.

Number	Compound	RI	RT	Structure	Molecular formula	References
1	Methyl isobutyl ketone	735	4.105	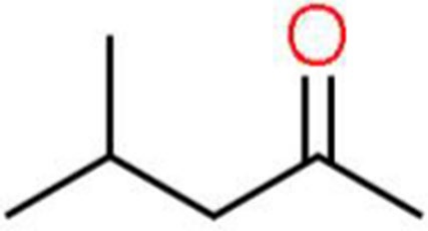	C_6_H_12_O	Han and Bhat (2014)
2	3,4,5-Trimethylpyrazole	1,100	11.025	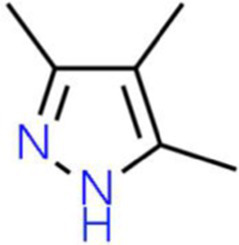	C_6_H_10_N_2_	Soltani et al. (2020)
3	Dimethyl trisulfide	970	11.298	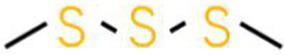	C_2_H_6_S_3_	Wu et al. (2023)
4	5-Hepten-2-one, 6-methyl-	986	12.162	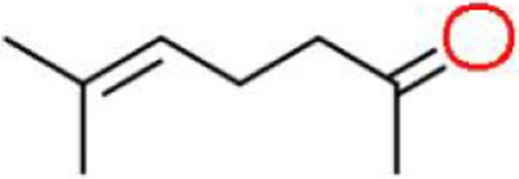	C_8_H_14_O	Bellahcen et al. (2019)
5	Pyridine, 2,4,6-trimethyl-	991	12.446	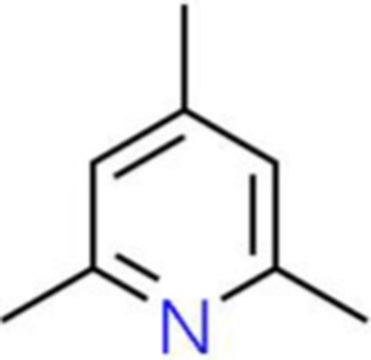	C_8_H_11_N	Wei et al. (2018)
6	Nonanal	1,104	17.822	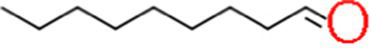	C_9_H_18_O	Fan et al. (2020)
7	Decanal	1,206	22.651	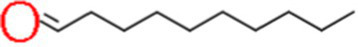	C_10_H_20_O	Fujita et al. (2015)
8	Dodecanal	1,409	31.732	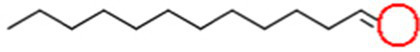	C_12_H_24_O	Ferreira et al. (2023)
9	2,4-Di-tert-butylphenol	1,519	35.776	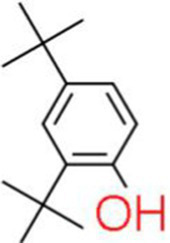	C_14_H_22_O	Seenivasan et al. (2022)
10	Dibutyl phthalate	1965	45.201	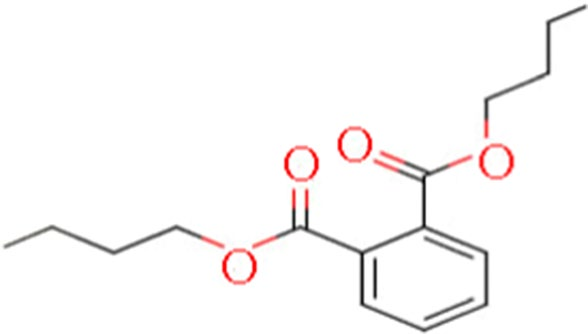	C_16_H_22_O_4_	Bi et al. (2021)

**Table 2:** Antimicrobial active components identified from CFS of LMY3-5 by LC–MS analysis.

Number	Compound	RT	Structure	Molecular formula	References
1	Isobutyric acid	0.942	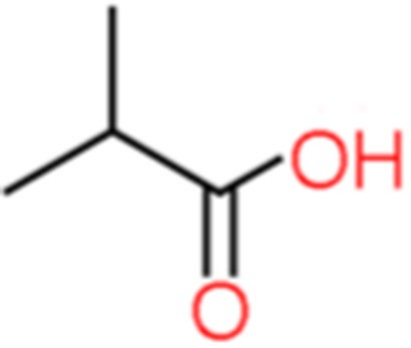	C_4_H_8_O_2_	Choi et al. (2013)
2	Acetamidobutanoic acid	0.77	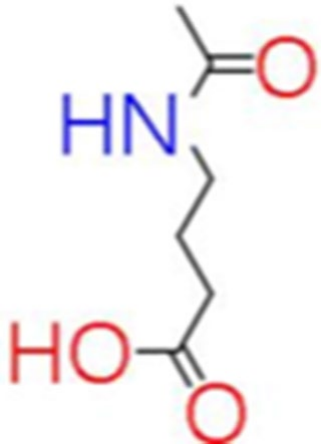	C_6_H_11_NO_3_	Xing et al. (2020)
3	Hydroxybenzaldehyde	5.746	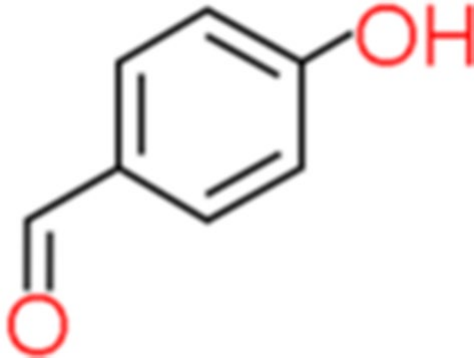	C_7_H_6_O_2_	Almenar et al. (2007)
4	Salicylic acid	4.308	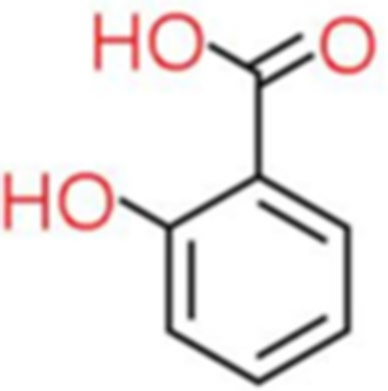	C_7_H_6_O_3_	Ma et al. (2023)
5	Trans-Cinnamic acid	3.574	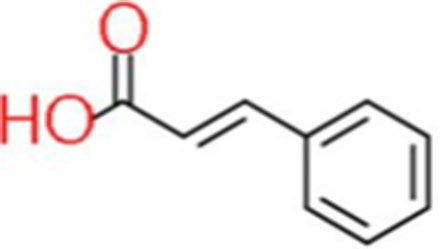	C_9_H_8_O_2_	Li et al. (2023)
6	Vanillin	7.451	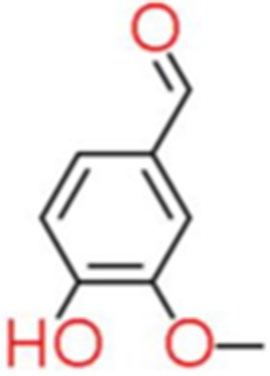	C_8_H_8_O_3_	Yang et al. (2021)
7	6-Gingerol	4.167	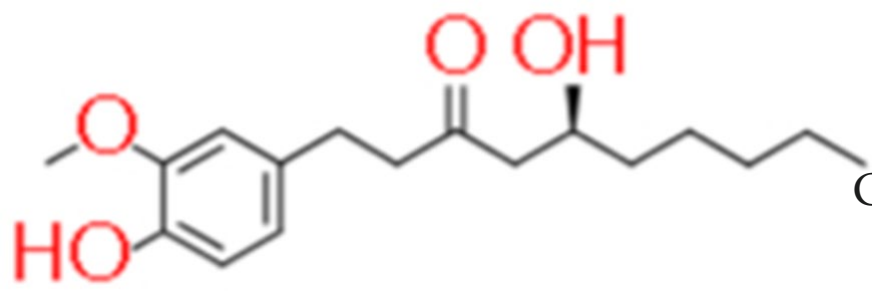	C_17_H_26_O_4_	dos Santos et al. (2023)
8	Guanidinobutyric acid	17.359	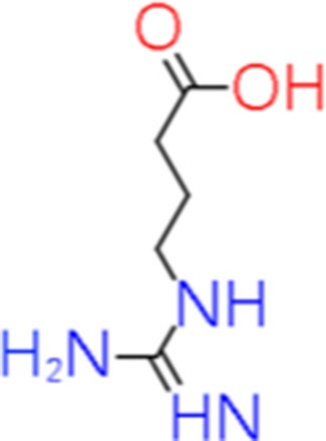	C_5_H_11_N_3_O_2_	Hwang and Jeong (2012)
9	Hydroxycinnamic acid	2.702	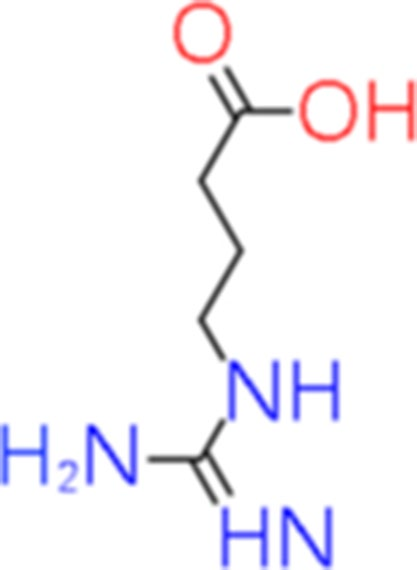	C_9_H_8_O_3_	Deniz and Ferda (2019)
10	Indolecarbaldehyde	4.495	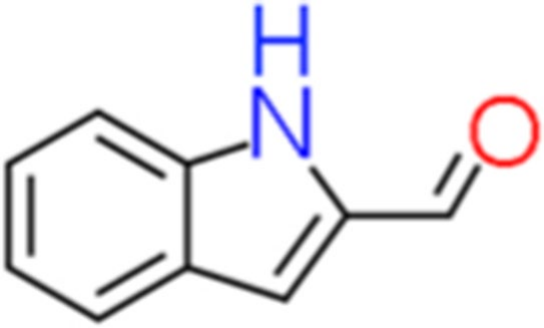	C_9_H_7_NO	Priya et al. (2022)
11	Betaine	0.697	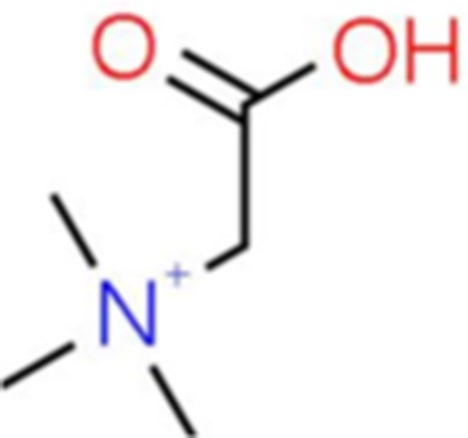	C_5_H_12_NO_2_	Blagodatskikh et al. (2018)
12	3-Phenyllactic acid	7.97	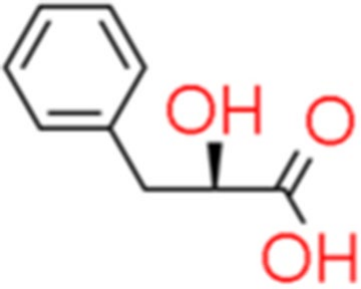	C_9_H_10_O_3_	Liu et al. (2021)
13	Oleamide	17.359	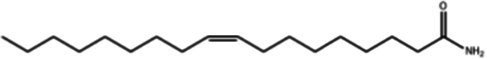	C_18_H_35_NO	Peng et al. (2023)
14	Bis(2-ethylhexyl) phthalate	19.59	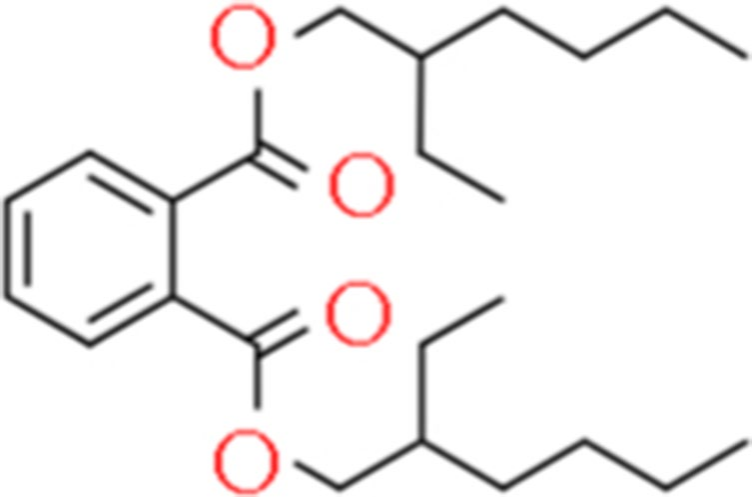	C_24_H_38_O_4_	Islam et al. (2013)
15	Dibutyl phthalate	15.067	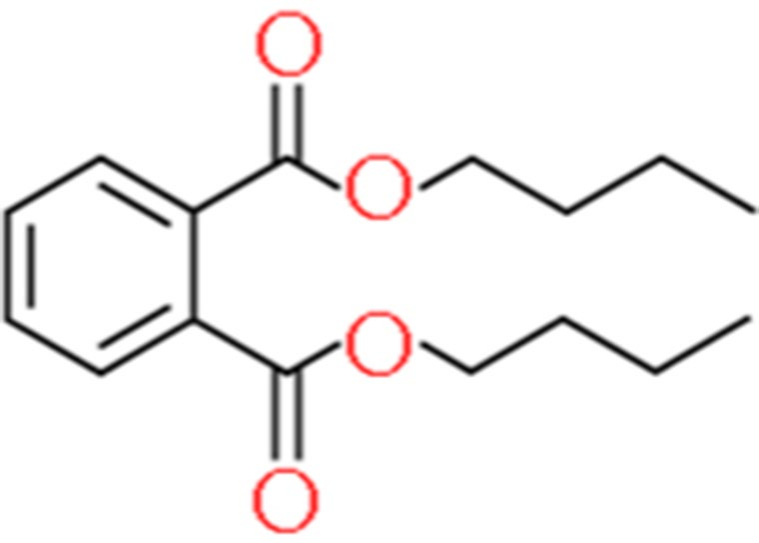	C_16_H_22_O_4_	Rajamanikyam et al. (2017)
16	8-Hydroxyquinoline	4.664	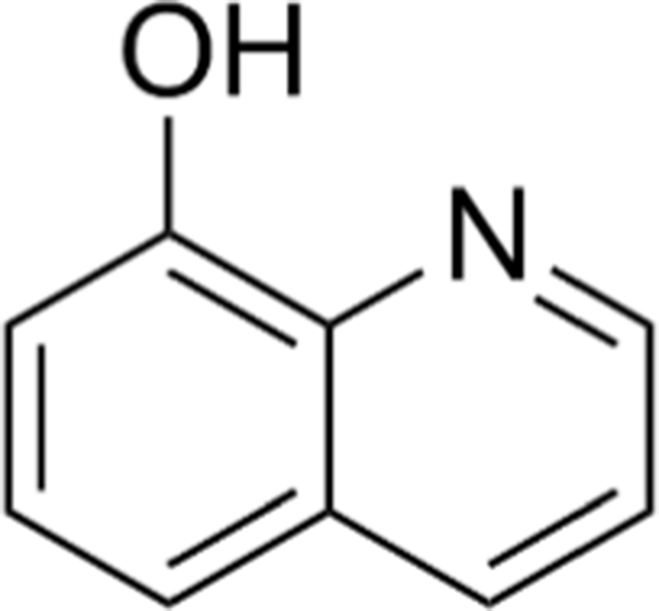	C_9_H_7_NO	Gupta et al. (2021)
17	Erucamide	18.226	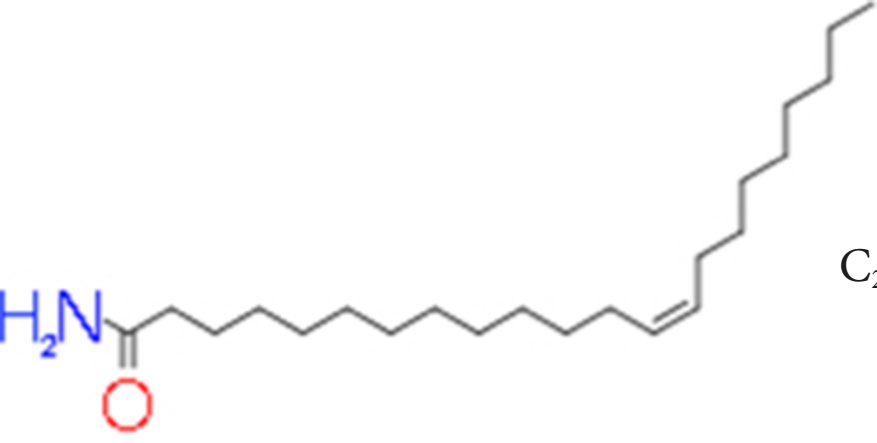	C_22_H_43_NO	Xie et al. (2021)

## 4 Discussion

Kiwifruit soft rot severely constrains the development of the kiwi industry. Currently, the prevention and control of kiwifruit soft rot primarily rely on chemical agents. In this study, the *B. velezensis* strain LMY3-5 was screened from the endophytes of kiwifruit and found to exhibit a marked inhibitory effect on the pathogen *B. dothidea*, which causes kiwifruit soft rot. *B. velezensis* has been confirmed to possess antimicrobial activity and can effectively suppress diseases such as peach gummosis (Kang et al., 2024), apple canker (Yuan et al., 2022), and pear ring rot (Yang et al., 2023). However, few studies have reported on the control effect of *B. velezensis* on kiwifruit soft rot. In this study, we demonstrated that *B. velezensis* LMY3-5 can effectively inhibit the expansion of kiwifruit soft rot. This is the first time that *B. velezensis* with biocontrol properties has been isolated from the endophytes of kiwifruit. Thus, our findings enrich the resource library for the biological control of kiwifruit soft rot and offer new possibilities for the biological control of this disease.

*B. velezensis* has a broad antifungal spectrum and is non-pathogenic to plants (Liu et al., 2017). The antimicrobial action in *bacillus* spp. is the most critical, and it is achieved by the production of antibiotic compounds, such as lipopeptides, peptides, bacteriocins, and volatile substances (Shafi et al., 2017; Fira et al., 2018). These substances inhibit pathogenic fungal hyphae growth and spore germination by acting on the fungal cell wall, cell membrane, and nuclear membrane and inducing apoptosis (Ramarathnam et al., 2007; Gu et al., 2017; Jin et al., 2020). In this experiment, the sterile fermentation broth of *B. velezensis* LMY3-5 affected *B. dothidea* by inhibiting hyphal growth and spore germination, damaging the cell membrane and cell wall, altering the morphology of the fungal body, increasing the hyphal permeability, and causing leakage of the nucleic acid content, thus leading to an imbalance of the intracellular environment and even cell death. These results are consistent with previous studies, suggesting that the *B. velezensis* strain LMY3-5 can produce multiple antimicrobial substances. However, the specific mechanisms of action and metabolic products remain to be elucidated in further experimental research.

The genus *Bacillus* is metabolically vigorous and capable of producing a variety of antimicrobial organic compounds, which can be broadly categorized into aldehydes, ketones, alcohols, esters, and organic acids (Zheng et al., 2013). However, even within the same species, different strains of *Bacillus* can produce varying antimicrobial compounds (Aloo et al., 2019). This study aims to preliminarily reveal the types of antimicrobial organic compounds produced by *B. velezensis* LMY3-5 by analyzing the components of its sterile filtrate using GC–MS and LC–MS techniques. The study found that the sterile filtrate contained 27 antifungal compounds, including isobutyric acid, dibutyl phthalate, nonanal, decanal, vanillin, and 2,4-di-tert-butylphenol. Previous reports have confirmed the antifungal capabilities of these substances. Aldehyde substances such as nonanal, decanal, and vanillin can inhibit the growth of the mycelium of *A. alternata*, *Penicillium*, *Botrytis cinerea*, and *Sclerotinia sclerotiorum* (Yang et al., 2021; Zhang et al., 2017; Zhang et al., 2013). The ketone methyl isobutyl ketone and ester dibutyl phthalate have strong antifungal activity against *Penicillium italicum* and *B. cinerea*, respectively (Morita et al., 2019; Li et al., 2022). Isobutyric acid has a broad spectrum of antimicrobial activity and also exhibits antibacterial activity against a variety of oral microorganisms (Huang et al., 2011). 4-Guanidinobutyric acid inhibits the growth of *Helicobacter pylori* and shows cytotoxicity to human gastric cancer cell lines SNU638 and AGS; thus, it can be used for the treatment and prevention of gastric damage (Hwang and Jeong, 2012). The presence of these compounds not only confirms the potential of *B. velezensis* LMY3-5 as a biocontrol agent but also reveals further possibilities for its application in the pharmaceutical industry.

In this experiment, *B. velezensis* LMY3-5 showed significant biocontrol potential and inhibited the expansion rates of *B. dothidea*, the causative agent of kiwifruit soft rot, by 73.59%. However, the efficacy of this strain in controlling kiwifruit soft rot under field conditions still needs to be evaluated through further field trials. Moreover, this study conducted a preliminary investigation into the antifungal mechanism of this strain at the cellular level. Future research could combine transcriptomics, proteomics, metabolomics, and multi-omics analyses to delve deeper into the molecular mechanisms of action, with the aim of providing theoretical support for the development of more comprehensive and efficient biocontrol strategies.

## Data Availability

The original contributions presented in the study are included in the article/supplementary material, further inquiries can be directed to the corresponding author.
